# Haemophilus influenzae type b and pneumococcal conjugate vaccination coverage in children aged 2-59 months in Malawi

**DOI:** 10.1080/21645515.2020.1773142

**Published:** 2020-06-23

**Authors:** Daphne Gadama, Tisungane Mvalo, Amy Sarah Ginsburg

**Affiliations:** a University of North Carolina Project Malawi, Lilongwe, Malawi; b Department of Pediatrics, School of Medicine, University of North Carolina at Chapel Hill, Chapel Hill, NC, USA; c University of Washington Clinical Trial Center, Seattle, WA, USA

**Keywords:** Pneumococcal vaccine, vaccination, pneumonia, children

## Abstract

High vaccination coverage with *Haemophilus influenzae* type b (Hib) and pneumococcal conjugate vaccines is critical to addressing childhood pneumonia’s high mortality. The Innovative Treatments in Pneumonia project in Lilongwe, Malawi collected Hib and pneumococcal vaccination information from children’s health passports and found the majority demonstrated inadequate and inconsistent documentation. We were unable to confidently assess the impact of Hib and pneumococcal vaccination on pneumonia treatment failure in our study population. Whether it be that enrolled children did not receive age-eligible vaccines or there was poor vaccination record-keeping, we do not know. A shift to an electronic data collection system may help ensure capture of essential vaccination data and provide more accurate records of both individual and population vaccination coverage in Malawi.

Pneumonia remains the leading infectious cause of death in children worldwide.^1^ Prevention of pneumonia through vaccination with *Haemophilus influenzae* type b (Hib) and *Streptococcus pneumoniae* conjugate vaccines is critical to addressing pneumonia’s high mortality. Yet, realizing the preventative and life-saving potential of vaccination requires high coverage. In many countries, vaccine coverage rates are below targets established by international and national advisory committees.^2^ The Global Vaccine Action Plan 2011–2020, endorsed by the World Health Assembly in 2012, calls on all countries to reach ≥90% national coverage for all vaccines in their routine vaccination schedules by 2020.^3^ This is not going to happen by the close of this year, especially due to the effect of the COVID-19 pandemic causing extended interruptions in vaccination programs worldwide.

In Africa, vaccination coverage for diphtheria, tetanus and pertussis vaccine dose 3 (DTP3) increased from 57% in 2000 to 76% in 2015, still lower than expected despite improved coverage.^4^ In Malawi, the proportion of children who received full vaccination before their first birthday dropped from 81% to 76% between 2010 and 2015; thus, leaving the remainder unvaccinated or under-vaccinated.^5^

Hib conjugate vaccine (as part of the pentavalent diphtheria, pertussis, tetanus, Hib and hepatitis B vaccine) and 13-valent pneumococcal conjugate vaccine (PCV13) were introduced in Malawi in January 2002 and November 2011, respectively using a three-dose primary series at 6, 10, and 14 weeks of age. In an assessment of PCV13 vaccine coverage using a repeated cross-sectional household survey to longitudinally track pediatric PCV13 coverage in rural Lilongwe, Malawi, a total of 8,562 children were surveyed in 6 surveys, and showed that despite large increases in two- and three-dose coverage among age-eligible children between March 2012 and June 2014, PCV13 coverage was consistently delayed and did not meet the Malawi Ministry of Health one-year three-dose 90% coverage target.^6^ This is in contrast to the Malawi Ministry of Health national vaccination coverage reports which showed the percentage of surviving infants who received the third doses of the Hib conjugate vaccine and PCV13 in 2018 to be 92% for both these vaccines.^7^

From 2016 to 2019, the Innovative Treatments in Pneumonia (ITIP) clinical studies were conducted in Lilongwe, Malawi at Kamuzu Central and Bwaila District Hospitals with the goal of assessing the optimal duration of treatment with amoxicillin for childhood pneumonia.^8–10^ A total of 5,127 children aged 2 to 59 months with pneumonia were enrolled and followed for 14 days with treatment failure as the primary outcome. Baseline data collected included childhood Hib conjugate and PCV13 vaccination information from enrolled children’s health passports (Table 1). In our cohort, age-eligible vaccination three-dose coverage was 61.2% for Hib conjugate vaccine and 60.9% for PCV13. The majority of enrolled children’s health passports demonstrated inadequate and inconsistent documentation, and we were unable to confidently assess the impact of Hib and pneumococcal vaccination on pneumonia treatment failure or Day 14 clinical cure rates in this study population. However, preliminarily, we did not find statistically significant differences in either treatment failure rates or clinical cure rates by Day 14 when comparing enrolled children who could be confirmed to have received at least two age-appropriate dosages of each vaccine compared to enrolled children who could not be confirmed to have received these dosages.

**Table 1.** Vaccination rates across ITIP studies

	ITIP1 N (%)			ITIP2 N (%)			ITIP3 N (%)			Total N (%)		
	<10 weeks	10–14 weeks	≥14 weeks	<10 weeks	10–14 weeks	≥14 weeks	<10 weeks	10–14 weeks	≥14 weeks	<10 weeks	10–14 weeks	≥14 weeks
	n = 5	n = 39	n = 1082	n = 81	n = 246	n = 2673	n = 35	n = 111	n = 855	n = 121	n = 396	n = 4610
Pentavalent vaccine (includes *Haemophilus influenzae* type b conjugate vaccine)												
Received age-appropriate number of doses^a^, n (%)	5 (100%)	24 (61.5%)	620 (57.3%)	72 (88.9%)	132 (53.7%)	1697 (63.5%)	29 (82.9%)	50 (45%)	509 (59.5%)	106 (87.6%)	206 (52%)	2826 (61.3%)
All doses unknown, n (%)	0 (0%)	1 (2.6%)	411 (38%)	5 (6.2%)	2 (0.8%)	710 (26.6%)	1 (2.9%)	3 (2.7%)	242 (28.3%)	6 (5%)	6 (1.5%)	1363 (29.6%)
Some doses unknown, n (%)												
Received 2 doses, n (%)	0 (0%)	0 (0%)	45 (4.2%)	0 (0%)	0 (0%)	214 (8%)	0 (0%)	0 (0%)	77 (9%)	0 (0%)	0 (0%)	336 (7.3%)
Received 1 dose, n (%)	0 (0%)	14 (35.9%)	3 (0.3%)	0 (0%)	106 (43.1%)	43 (1.6%)	0 (0%)	49 (44.1%)	24 (2.8%)	0 (0%)	169 (42.7%)	70 (1.5%)
Received 0 doses, n (%)	0 (0%)	0 (0%)	3 (0.3%)	4 (4.9%)	6 (2.4%)	9 (0.3%)	5 (14.3%)	9 (8.1%)	3 (0.4%)	9 (7.4%)	15 (3.8%)	15 (0.3%)
Pneumococcal conjugate vaccine												
Received age-appropriate number of doses^a^, n (%)	5 (100%)	24 (61.5%)	619 (57.2%)	72 (88.9%)	133 (54.1%)	1689 (63.2%)	28 (80%)	50 (45%)	503 (58.8%)	105 (86.8%)	207 (52.3%)	2811 (61%)
All doses unknown, n (%)	0 (0%)	1 (2.6%)	412 (38.1%)	5 (6.2%)	2 (0.8%)	712 (26.6%)	1 (2.9%)	3 (2.7%)	242 (28.3%)	6 (5%)	6 (1.5%)	1366 (29.6%)
Some doses unknown, n (%)												
Received 2 doses, n (%)	0 (0%)	0 (0%)	46 (4.3%)	0 (0%)	0 (0%)	219 (8.2%)	0 (0%)	0 (0%)	82 (9.6%)	0 (0%)	0 (0%)	347 (7.5%)
Received 1 dose, n (%)	0 (0%)	14 (35.9%)	2 (0.2%)	0 (0%)	105 (42.7%)	44 (1.6%)	0 (0%)	49 (44.1%)	25 (2.9%)	0 (0%)	168 (42.4%)	71 (1.5%)
Received 0 doses, n (%)	0 (0%)	0 (0%)	3 (0.3%)	4 (4.9%)	6 (2.4%)	9 (0.3%)	6 (17.1%)	9 (8.1%)	3 (0.4%)	10 (8.3%)	15 (3.8%)	15 (0.3%)

^a^– One dose if at least 6 weeks and up to 10 weeks old, two doses if at least 10 weeks and up to 14 weeks old, three doses if at least 14 weeks old.

Similar to the 2012–2014 findings from the Lilongwe cross-sectional household survey,^6^ our data show lower reported vaccination rates among enrolled ITIP children compared to the data from the Malawi Ministry of Health national vaccination coverage reports.^7^ Differences in methods and systems of data collection could contribute to these differences. For the ITIP vaccination data, a dose of a vaccine was only recorded as received if documentation of the vaccination was noted in the child’s health passport; otherwise, it was recorded by the ITIP study team as unknown. Other reports use caregiver verbal reports^6^ or facility-level data that report the number of doses of vaccines administered per facility.^7^ A 2018 GAVI Joint Appraisal report for Malawi’s Expanded Program on Immunization noted that registers at vaccination sites were not user-friendly, resulting in poor data capture and data loss.^11^ Of note, another study from Malawi has shown that children with no health passport or whose health passport was lost have an increased odds of being both non- and under-vaccinated.^5^

Beyond the lack of or poor vaccination documentation or reporting issues, low vaccine uptake and untimely vaccination among the enrolled ITIP children could be due to a variety of factors including but not limited to low socioeconomic status, low maternal education, non-facility birth, and increased distance to a health facility.^5,12^ Missed opportunities for vaccination during healthcare visits also contribute to unfulfilled childhood vaccination targets.^13^ In some facilities in Malawi, reported vaccination coverage was less than 50% and was attributed to a lack of awareness, knowledge or prioritization by healthcare providers and caregivers, poor coordination and referral of eligible children, and unavailability of vaccines.^13^ Higher vaccination rates in the older age group of enrolled ITIP children could suggest delayed or late administration of vaccines due to lack of adequate vaccine supplies, lack of trained healthcare providers to administer the vaccines, or delayed care-seeking by caregivers.

Malawi has worked hard to provide timely and universal vaccination throughout the country and has been a regional leader in ensuring expanded vaccination coverage.^12^ The data from our ITIP project is limited in that we used only the health passport documentation as proof of Hib conjugate vaccine and PCV13 vaccination. Despite not having the appropriate documentation, children in our studies may have received appropriate vaccination. We do not know. Nevertheless, whether it be poor documentation or that they did not receive the age-eligible vaccines, gaps remain. A shift to an electronic data collection system may help to ensure capture of essential vaccination data and provide a more accurate record of both individual and population vaccination coverage in Malawi. Challenges around access to vaccination services and healthcare persist, and joint efforts between the government and key stakeholders continue to be critical in combatting vaccine preventable disease and death.
